# Isolated leptomeningeal angiomatosis in Sturge-weber syndrome type III: A case report with distinctive neuroimaging features

**DOI:** 10.1016/j.radcr.2025.01.092

**Published:** 2025-03-09

**Authors:** Azzeddine Laaraje, Salah Ben elhend

**Affiliations:** a Department of Pediatrics, Mohammed V Military Training Hospital, Rabat, Morocco; b Department of Radiology, Avicenne Military Hospital, Marrakech, Morocco

**Keywords:** Sturge-Weber syndrome type III, Leptomeningeal angiomatosis, Neuroimaging, Drug-resistant epilepsy, Cortical calcifications, Magnetic resonance imaging

## Abstract

Sturge-Weber syndrome type III (SWS-III) is the rarest variant of SWS, characterized by isolated leptomeningeal angiomatosis without cutaneous or ophthalmological manifestations. We report the case of an 11-year-old female who developed drug-resistant epilepsy at 18 months of age and mild left hemiparesis. Despite multiple anticonvulsant medications, seizures remained poorly controlled. Brain imaging revealed characteristic findings of right cerebral hemihypotrophy, cortical calcifications, and leptomeningeal enhancement without facial port-wine stain or ophthalmological involvement. These distinctive radiological features led to the diagnosis of SWS-III. This case highlights the crucial role of neuroimaging in diagnosing SWS-III, where external clinical markers are absent. Recognition of specific radiological patterns is essential for accurate diagnosis and appropriate management.

## Introduction

Sturge-Weber syndrome (SWS) is a rare neurocutaneous disorder with an estimated prevalence between 1/20,000 and 1/50,000 live births [1,2]. The classical form is characterized by leptomeningeal angiomas, typically associated with facial port-wine stains and ocular anomalies. These manifestations form the traditional diagnostic triad. Type III SWS, the rarest variant representing less than 10% of cases, presents with isolated leptomeningeal involvement without cutaneous or ocular manifestations, making radiological findings crucial for diagnosis [3,4]. In 2013, a breakthrough discovery revealed that SWS results from a somatic mosaic activating R183Q mutation in the GNAQ gene. This mutation disrupts vascular development through constitutive activation of the RAS-MEK-ERK pathway, leading to characteristic neuroimaging features including leptomeningeal enhancement, cortical calcifications, and cerebral atrophy [2,5]. These radiological hallmarks become particularly critical diagnostic markers in Type III SWS where external clinical manifestations are absent. The neuroimaging pattern in SWS presents 2 distinct phenotypes: predominant vascular anomalies (characterized by leptomeningeal angiomas and dilated deep veins) in approximately 60% of cases, and predominant parenchymal involvement (marked by atrophy and cortical calcifications) in the remaining 40% [6]. We present here a case of Type III SWS demonstrating the second phenotype, which illustrates how specific neuroimaging patterns can guide diagnosis and potentially inform prognosis in the absence of classical clinical markers.

## Case report

**Fig. 1:**
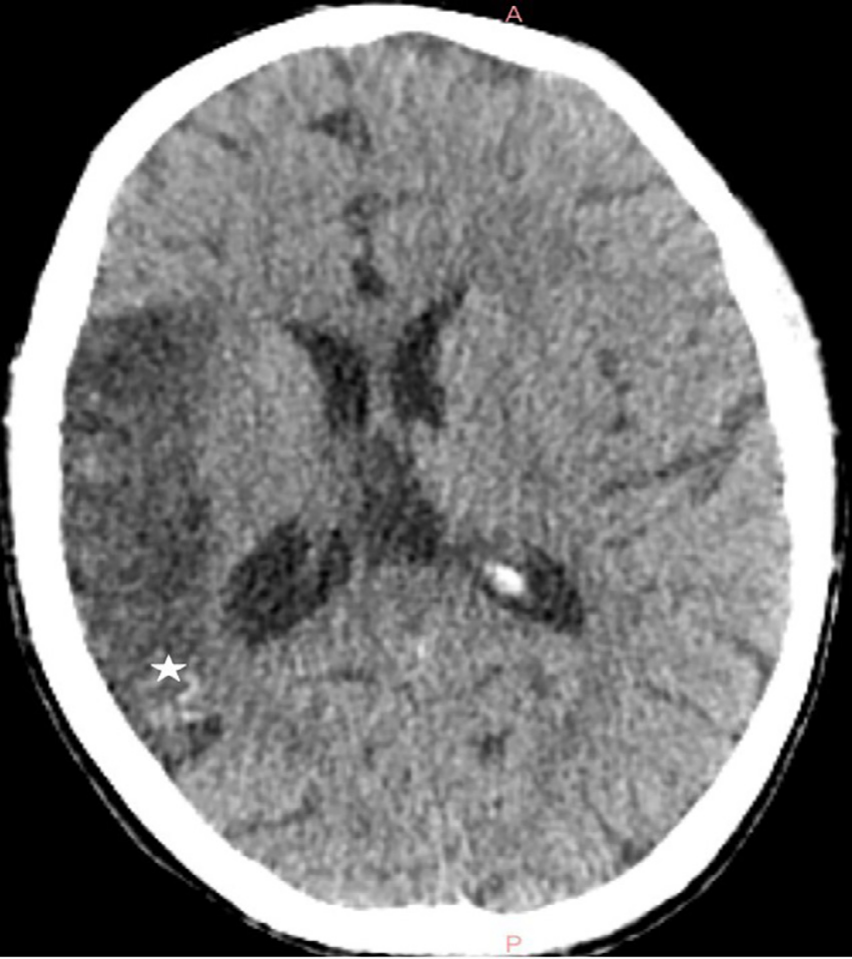
Axial noncontrast brain CT in Sturge-Weber syndrome type III The image shows right cerebral hemihypotrophy with spontaneous cortical hyperdensities corresponding to fine calcifications (*). These gyriform calcifications are characteristic of SWS and represent chronic hypoxic injury to the cortex.

**Fig. 2:**
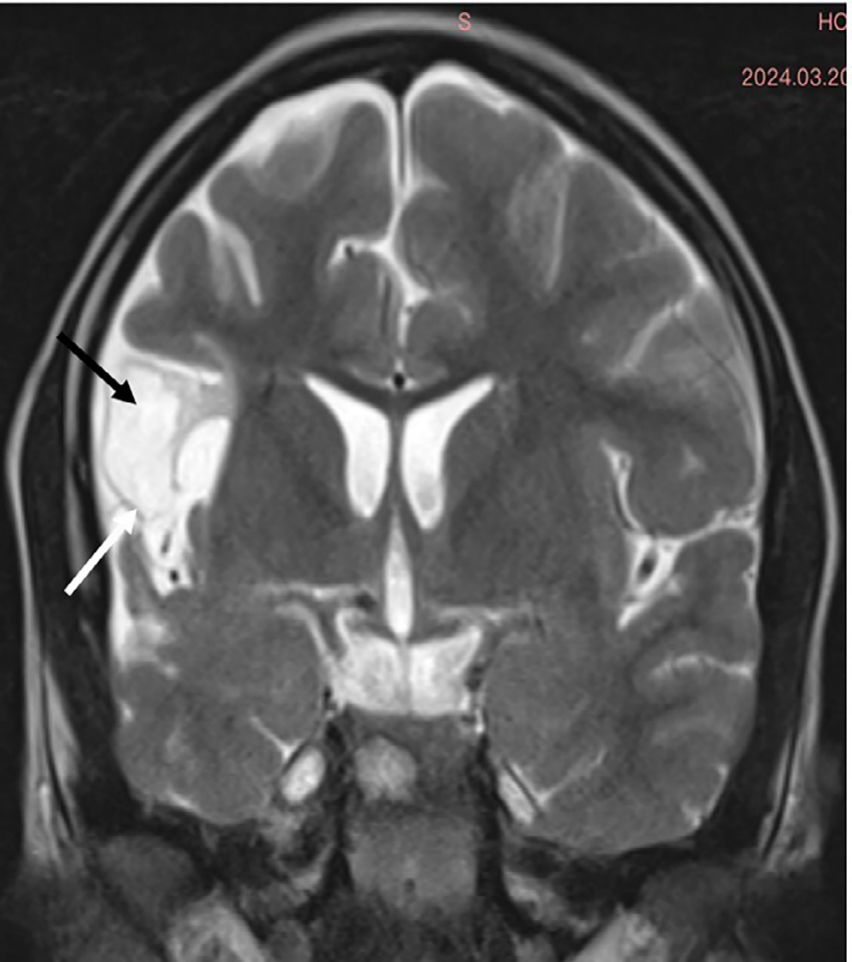
Coronal FLAIR MRI in Sturge-Weber syndrome type III. The image demonstrates hemiatrophy of the right cerebral hemisphere with prominent involvement of temporal and parietal lobes (black arrow), associated with congested veins (white arrow). These findings reflect chronic hypoperfusion and venous congestion typical of SWS.

**Fig. 3:**
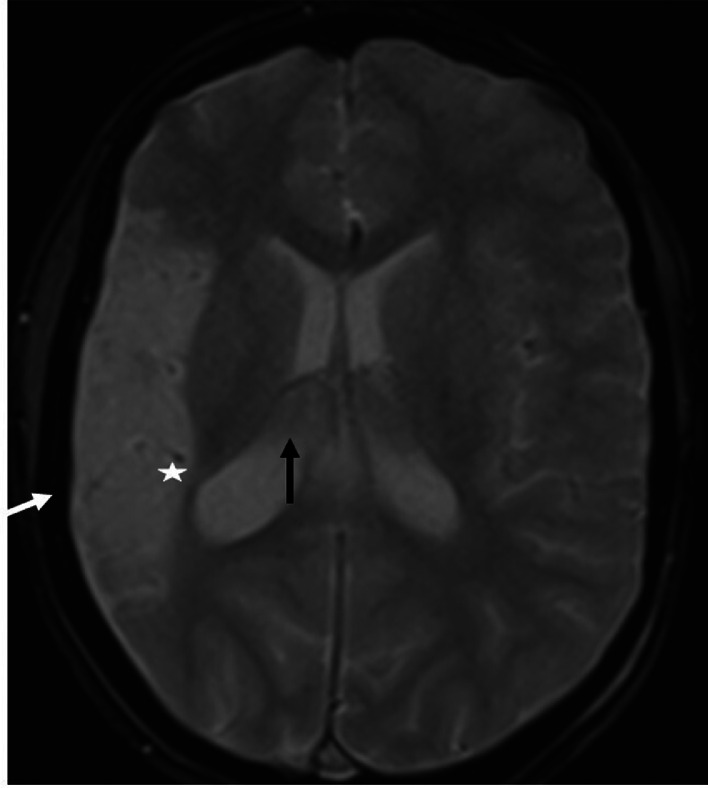
Axial T2* MRI in Sturge-Weber syndrome type III The sequence reveals dilatation of transparenchymal veins (*) with enlarged ipsilateral choroid plexus (black arrow) and calvarial thickening (white arrow). These features represent the characteristic parenchymal pattern of SWS type III.

An 11-year-old female patient, born from a first-degree consanguineous marriage, was followed for attention deficit hyperactivity disorder (ADHD) and drug-resistant epilepsy. Her birth was marked by neonatal asphyxia during a home delivery, with 4 minutes of anoxia and an Apgar score of 4 at 1 minute and 7 at 5 minutes. Early childhood was notable for delayed psychomotor development with walking acquired at 20 months. The patient began experiencing focal motor seizures affecting the left hemibody at 18 months of age, initially occurring weekly. These seizures remained poorly controlled despite various therapeutic combinations including sodium valproate (Depakine®), carbamazepine (Tegretol®), and levetiracetam (Keppra®), with a current frequency of 1 seizure every 15 days. Physical examination revealed a mild left hemiparesis rated 4/5, predominantly affecting the upper limb. Growth parameters were slightly below normal with a weight of 35 kg (-1 SD) and height of 142 cm (-0.5 SD). Neuropsychological evaluation showed a global IQ of 85 and a 2-year learning delay. Notably, cutaneous examination showed no port-wine stains, and ophthalmological examination was strictly normal, with no glaucoma or choroidal hemangioma. Radiological investigations revealed distinctive features. Brain CT without contrast showed right cerebral hemihypotrophy with spontaneous cortical hyperdensities corresponding to fine calcifications (Fig. 1). Brain MRI performed on a 3-Tesla scanner demonstrated on coronal FLAIR sequences right cerebral hemisphere hemiatrophy, particularly prominent in temporal and parietal regions, with presence of congested veins (Fig. 2). Axial T2* sequences revealed dilatation of transparenchymal veins, enlargement of ipsilateral choroid plexus, and calvarial thickening (Fig. 3). MR angiography confirmed hypoperfusion of the affected hemisphere without major vascular anomaly. EEG showed focal epileptic activity in the temporal right region, with isolated spikes and waves bursts during sleep. Based on these clinical, electrophysiological, and particularly radiological findings showing the characteristic triad of hemiatrophy, calcifications, and leptomeningeal enhancement without cutaneous or ophthalmological involvement, a diagnosis of Sturge-Weber syndrome type III was established.

## Discussion

In this case report, we present the radiological features of Sturge-Weber Syndrome (SWS) type III, a rare form characterized by isolated leptomeningeal involvement without cutaneous or ophthalmological manifestations, representing approximately 10% of SWS cases [1,3]. The main interest of this case lies in its distinctive neuroradiological characteristics, which perfectly illustrate the parenchymal phenotype of SWS. Recent studies, notably by Hadjinicolaou et al. [3], have established 2 distinct radiological patterns: a predominant vascular pattern characterized by leptomeningeal angiomas and dilated deep veins, and a predominant parenchymal pattern marked by atrophy and cortical calcifications [6]. Our case clearly demonstrates the second pattern, with typical CT findings showing cerebral hemiatrophy and characteristic cortical calcifications. The calcifications typically follow a gyral pattern and are best visualized on noncontrast CT, appearing as linear or curvilinear hyperdensities in the affected cortex. The MRI sequences allowed for more precise characterization of the abnormalities. Coronal FLAIR sequences highlighted right cerebral hemiatrophy, particularly prominent in temporal and parietal regions, with associated white matter signal changes reflecting chronic hypoxic-ischemic injury. T2* sequences were particularly valuable in demonstrating transparenchymal venous dilatation, ipsilateral choroid plexus enlargement, and calvarial thickening. Postcontrast sequences, although not performed in our case, typically show leptomeningeal enhancement with a characteristic “tram-track” pattern due to contrast enhancement of engorged pial vessels. These radiological features are pathognomonic of SWS type III and allow for definitive diagnosis in the absence of external clinical signs [3,4]. A significant point demonstrated by our case is the reliability of noncontrast brain CT for initial diagnosis, as reported in previous studies [7]. The presence of characteristic gyriform cortical calcifications on CT, typically appearing by age 2, can be sufficient to suggest the diagnosis. These calcifications represent chronic hypoxic injury to the cortex underlying the leptomeningeal angioma. While contrast-enhanced MRI remains the reference examination for evaluating the exact extent of leptomeningeal involvement [3,4], CT findings can be diagnostic when they show the characteristic pattern of calcifications. MR angiography provided complementary information by demonstrating hypoperfusion of the affected hemisphere, an element that may have prognostic value. The chronic venous congestion leads to progressive parenchymal volume loss and increased collateral venous drainage, visible on MRI. Recent studies have shown that the degree of cerebral perfusion asymmetry correlates with clinical severity [8]. Advanced MRI techniques such as susceptibility-weighted imaging (SWI) and perfusion studies can provide additional valuable information about the extent of venous abnormalities and perfusion deficits. From an imaging perspective, our case also illustrates the temporal evolution of radiological findings in SWS [9]. The presence of both calcifications and atrophy suggests a chronic process, as calcifications typically develop in early childhood while atrophy represents the cumulative effect of chronic hypoperfusion. The recognition of these temporal patterns is crucial for accurate diagnosis and prognosis assessment.

## Declaration of generative AI and AI-assisted technologies in the writing process

During the preparation of this work the author(s) used Claude in order to improve readability and English language editing. After using this tool, the author(s) reviewed and edited the content as needed and take(s) full responsibility for the content of the publication.

## Author contributions

**AL:** Study conception, data collection, manuscript writing. **SB:** Radiological analysis and interpretation, image preparation.

## Patient consent

I obtained a written informed consent from the patient for images or other clinical information relating to his case to be reported in a medical publication.

## References

[1] Siri L, Giordano L, Accorsi P, Cossu M, Pinelli L, Tassi L (2013). Clinical features of Sturge-Weber syndrome without facial nevus: five novel cases. Eur J Paediatr Neurol.

[2] Shirley MD, Tang H, Gallione CJ, Baugher JD, Frelin LP, Cohen B (2013). Sturge-Weber syndrome and port-wine stains caused by somatic mutation in GNAQ. N Engl J Med.

[3] Hadjinicolaou A, Quinlan A, Liu S, Zhang B, Takeoka M, Sahin M (2024). Variation in neuroimaging and outcomes in patients with Sturge Weber syndrome Type III. Brain Dev.

[4] Jordan PR, Iqbal M, Prasad M. (2016). Sturge-Weber syndrome type 3 manifesting as ‘Status migrainosus’. BMJ Case Rep.

[5] Yeom S, Comi AM. (2022). Updates on Sturge-Weber syndrome. Stroke.

[6] Niang I, Banfiteye A, Ndoye NA, Ba E, Diop O, Mbaye M (2020). Sturge Weber syndrome, when brain CT is enough for diagnosis: about a case. Pan Afr Med J.

[7] Jimenez-Legido M, Martinez-de-Azagra A, Bernardino B, Solis-Muniz I, Soto-Insuga V, Cantarin-Extremera V (2020). Utility of the transcranial doppler in the evaluation and follow-up of children with Sturge-Weber Syndrome. Eur J Paediatr Neurol.

[8] Muniz BC, Santos LR, Oliveira LAN, Marchiori E. (2022). Sturge-Weber syndrome with bilateral cerebral and facial involvement. Neurol India.

[9] Rubin A, Waszczuk Ł, Trybek G, Kapetanakis S, Bladowska J (2022). Application of susceptibility weighted imaging (SWI) in diagnostic imaging of brain pathologies – A practical approach. Clin Neurol Neurosurgery.

